# Cystic fibrosis sputum media induces an overall loss of antibiotic susceptibility in *Mycobacterium abscessus*

**DOI:** 10.1038/s44259-024-00054-3

**Published:** 2024-11-05

**Authors:** Emily J. Baker, Gemma Allcott, Antonia Molloy, Jonathan A. G. Cox

**Affiliations:** College of Health and Life Sciences, Aston University, Aston Triangle, Birmingham, B4 7ET UK

**Keywords:** Antibiotics, Infectious-disease diagnostics

## Abstract

*Mycobacterium abscessus* complex (MABSC) comprises a group of environmental microorganisms, which are a concerning cause of opportunistic respiratory infections in patients with cystic fibrosis or bronchiectasis. Only 45.6% of MABSC treatments are successful, and therefore this is a need to discover new antimicrobials that can treat these pathogens. However, the transferability of outcomes to the clinic is flawed by an inability to accurately represent the lung environment within the laboratory. Herein, we apply two preestablished formulations of sputum media (ACFS and SCFM1) to MABSC antibiotic susceptibility testing. Using conventional broth microdilution, we have observed strain and antibiotic dependent alterations in antimicrobial sensitivity in each sputum media compared standard laboratory media (7H9), with an overall reduction in susceptibility within the physiologically relevant conditions. We provide a timely contribution to the field of *M. abscessus* antibiotic discovery by emphasising the need for improved physiological relevance.

## Introduction

Antimicrobial resistance (AMR) is a major public health concern, which was associated with approximately 4.95 million deaths in 2019 alone^1^. One group of concerning pathogens includes the *Mycobacterium abscessus* complex (MABSC), comprising of three subspecies that can cause opportunistic respiratory infections in patients with cystic fibrosis. Namely, *M. abscessus* subsp. *abscessus*, *M. abscessus* subsp. *bolletii* and *M. abscessus* subsp. *massiliense* form MABSC, each of which demonstrate differential antimicrobial susceptibility profiles^2,3^. Although still considered emerging CF pathogens, *M. abscessus* is one of the most common species of rapidly growing mycobacteria identified worldwide^4^, effecting both adult and paediatric patients across the globe^5,6^. Despite this prevalence, the resistance exhibited by these species results in poor treatment success, with less than 50% of patients achieving bacterial clearance following antibiotic treatment^7^. This poor therapeutic success isn’t matched to the treatment’s intensity, with it involving a complex multidrug regimen typically lasting >12 months^8^. These challenges raise an urgent need to discover novel antimicrobials that can effectively clear *M. abscessus* species with minimal treatment burden, however the antibiotic discovery sector is flawed by its inability to accurately recapitulate the host environment. The complex behaviours and phenotypes adopted within the body are often responsible for a microbe’s resistance to treatment, and so underrepresentation of these conditions in vitro is likely to falsely portray antimicrobial susceptibility within the body. One important example includes the high concentrations of mucin found within CF sputum, with mucin having been previously implicated in AMR via antimicrobial binding^9^. With mucin concentrations 5–10 fold higher in CF sputum than that of healthy alternatives, there is the potential for CF sputum to be a driving force for in vivo resistance^10,11^; a factor which is rarely considered during in vitro drug screening. It is therefore unsurprising that treatment success remains so low, with our current in vitro assays being unpredictive of in vivo outcomes.

To overcome these challenges, two lineages of sputum mimetic media have been adopted within the last two decades: denoted artificial sputum media (ASM) and synthetic cystic fibrosis sputum media (SCFM). Both media types were initially designed to represent the nutritional components of sputum more closely and have been adapted through time to become as optimal as possible. The first to be published was ASM, derived by Ghani and Soothill in 1997 - based on the average concentrations of DNA, lipids, mucin, sodium, potassium and chloride within sputum^12^. This basic composition then faced a series of adaptations, including the addition of amino acids by Sriramulu et al. in 2005^13^ and Fung et al.’s addition of bovine serum albumin in 2010^14^. Conversely, SCFM1 is based on the average concentrations of glucose, free amino acids, iron and lactate found within CF sputum^15^—a formulation that was later adapted via the addition of DNA, mucin, dioleoylphosphatidylcholine and N-acetylglucosamine to form SCFM2^16^. These two lineages were combined in 2019 to form artificial cystic fibrosis sputum (ACFS); comprised of the components of ASM, plus 20 amino acids at concentrations equivalent to those used in SCFM1^17^.

Throughout time, each ASM and SCFM formulation has been utilised for a variety of different studies—however, these applications are predominantly to study the core CF pathogen *Pseudomonas aeruginosa*^13,14,16,18^. Aside from a few more recent publications^17,19,20^, little work has been done to apply these media types to the emerging CF pathogen *M. abscessus*. Given that the microenvironment of the cystic fibrosis lung has been shown to alter antibiotic activity in *P. aeruginosa*^21^, we sought address this knowledge gap by investigating MABSC antimicrobial susceptibility in Middlebrook 7H9 broth versus two types of sputum media—SCFM1^15^ and ACFS^17^. We selected 10 antibiotics that are routinely used in MABSC treatment (Section 2.2)^8,22,23^ in order to provide clinical representation. This data provides evidence to support the wider implementation of sputum media within antimicrobial discovery, therefore enhancing the clinical translation of future laboratory outcomes.

## Results

**Fig. 1:**
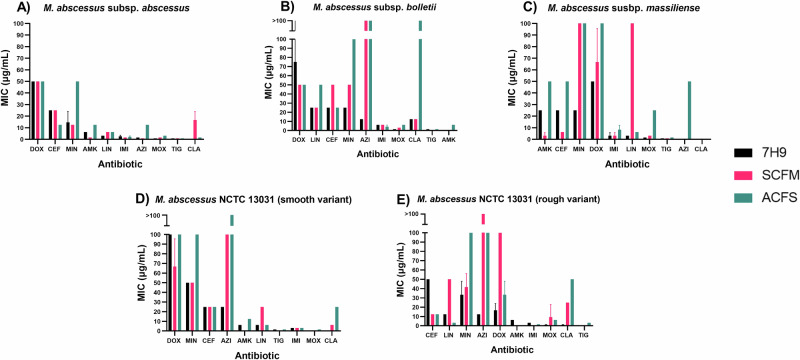
Minimum inhibitory concentration (MIC) of each antibiotic in each media. **A** *M. abscessus* subsp. *abscessus* (*n* = 3). **B** *M. abscessus* subsp. *bolletii* (*n* = 3). **C** *M. abscessus* subsp. *massiliense* (*n* = 3). **D** *M. abscessus* NCTC 13031 smooth variant (*n* = 3). **E** *M. abscessus* NCTC 13031 rough variant (*n* = 3). Error bars represent standard deviation. AMK amikacin, AZI azithromycin, CEF cefoxitin, CLA clarithromycin, DOX doxycycline, IMI imipenem, LIN linezolid, MIN minocycline, MOX moxifloxacin, TIG tigecycline.

**Fig. 2:**
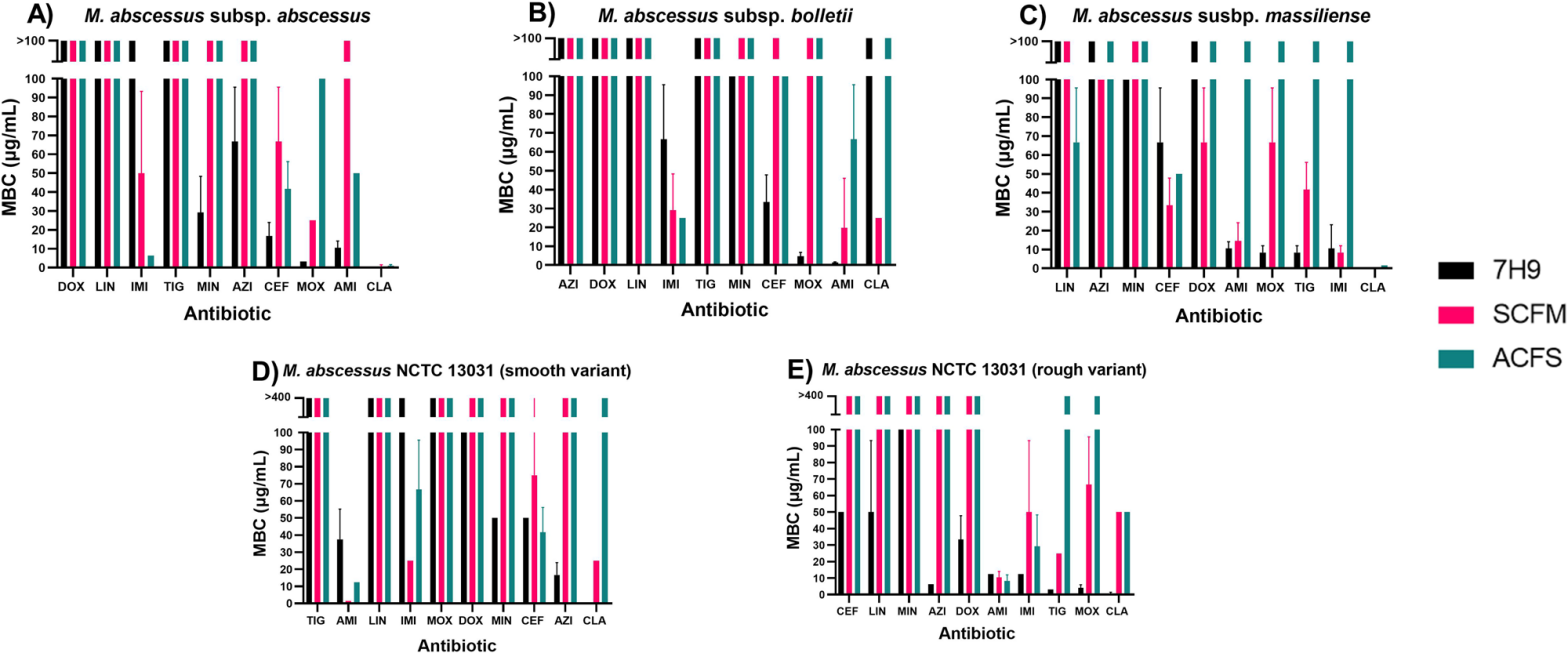
Minimum bactericidal concentration (MBC) of each antibiotic in each media. **A** *M. abscessus* subsp. *abscessus* (*n* = 3). **B** *M. abscessus* subsp. *bolletii* (*n* = 3). **C** *M. abscessus* subsp. *massiliense* (*n* = 3). **D** *M. abscessus* NCTC 13031 smooth variant (*n* = 3). **E** *M. abscessus* NCTC 13031 rough variant (*n* = 3). Error bars represent standard deviation. AMK amikacin, AZI azithromycin, CEF cefoxitin, CLA clarithromycin, DOX doxycycline, IMI imipenem, LIN linezolid, MIN minocycline, MOX moxifloxacin, TIG tigecycline.

To assess the impact of physiologically relevant media on MABSC antibiotic susceptibility, we have conducted a series of broth microdilution assays against five strains within MABSC, in both standard laboratory media (7H9) and comparative sputum media (ACFS and SCFM1). The MIC and MBC data from this analysis is depicted in Tables 1–5, and is colour coded in the form of greyscale heat maps in Supplementary Fig. 1. Although obvious differences were seen within each media, these discrepancies were largely strain and antibiotic dependent – therefore highlighting the unrepresented complexity of each clinical case. These differences have been displayed as bar charts in Figs. 1, 2, and in the form of fold change tables (Tables 6, 7) to quantify the differences between each media.

**Table 1:** Mean minimum inhibitory concentrations (MIC) and mean minimum bactericidal concentrations (MBC) of each drug in each media, against planktonic *M. abscessus* subsp. *abscessus*

M. *abscessus* subsp. *abscessus*						
Drug	7H9 + 10% ADC + 0.05% Tween 80		ACFS + 0.05% tyloxapol		SCFM1 + 0.05% tyloxapol	
	MIC (μg/mL)	MBC (μg/mL)	MIC (μg/mL)	MBC (μg/mL)	MIC (μg/mL)	MBC (μg/mL)
LINEZOLID	3.125	>100	6.25	>100	6.25	>100
MINOCYCLINE	14.58	29.16	50	>100	12.5	>100
DOXYCYLINE	50	>100	50	>100	50	>100
AMIKACIN	6.25	10.42	12.5	50	1.563	>100
CEFOXITIN	25	16.6	12.5	41.6	25	66.6
TIGEYCYLINE	0.78	>100	0.78	>100	0.78	>100
IMIPENEM	2.6	>100	2.08	6.25	1.563	50
AZITHROMYCIN	1.563	66.6	12.5	>100	0.78125	>100
CLARITHROMYCIN	0.098	0.098	1.56	0.911	16.6	0.618
MOXIFLOXACIN	0.78	3.125	3.125	100	1.563	25

**Table 2:** Mean minimum inhibitory concentrations (MIC) and mean minimum bactericidal concentrations (MBC) of each drug in each media, against planktonic *M. abscessus* subsp. *bolletii*

M. *abscessus* subsp. *bolletii*						
Drug	7H9 + 10% ADC + 0.05% Tween 80		ACFS + 0.05% tyloxapol		SCFM1 + 0.05% tyloxapol	
	MIC (μg/mL)	MBC (μg/mL)	MIC (μg/mL)	MBC (μg/mL)	MIC (μg/mL)	MBC (μg/mL)
LINEZOLID	25	>100	50	>100	25	>100
MINOCYCLINE	25	100	100	>100	50	>100
DOXYCYLINE	50	>100	50	>100	50	>100
AMIKACIN	0.391	1.953	6.25	66.6	0.098	19.79
CEFOXITIN	25	33.3	25	100	50	>100
TIGECYCLINE	1.56	>100	1.56	>100	0.39	>100
IMIPENEM	6.25	66.6	5.2	25	6.25	29.16
AZITHROMYCIN	12.5	>100	>100	>100	>100	>100
CLARITHROMYCIN	12.5	>100	>100	>100	12.5	25
MOXIFLOXACIN	1.5625	4.6875	6.25	>100	3.125	>100

**Table 3:** Mean minimum inhibitory concentrations (MIC) and mean minimum bactericidal concentrations (MBC) of each drug in each media, against planktonic *M. abscessus* subsp. *massiliense*

M. *abscessus* subsp. *massiliense*						
Drug	7H9 + 10% ADC + 0.05% Tween 80		ACFS + 0.05% tyloxapol		SCFM1 + 0.05% tyloxapol	
	MIC (μg/mL)	MBC (μg/mL)	MIC (μg/mL)	MBC (μg/mL)	MIC (μg/mL)	MBC (μg/mL)
LINEZOLID	3.125	>100	6.25	66.6	100	>100
MINOCYCLINE	25	100	100	>100	100	>100
DOXYCYLINE	50	>100	100	>100	66.6	66.6
AMIKACIN	25	10.42	50	>100	3.125	14.58
CEFOXITIN	25	66.6	50	50	6.25	33.3
TIGECYCLINE	0.78	8.3	1.56	>100	0.78	41.6
IMIPENEM	3.125	10.4	8.3	>100	9.375	8.3
AZITHROMYCIN	0.195	>100	50	>100	0.195	>100
CLARITHROMYCIN	0.098	0.098	0.391	1.563	0.0976	0.781
MOXIFLOXACIN	1.5625	8.3	25	>100	3.125	66.6

**Table 4:** Mean minimum inhibitory concentrations (MIC) and mean minimum bactericidal concentrations (MBC) of each drug in each media, against planktonic *M. abscessus* NCTC 13031 (smooth variant)

M. *abscessus* NCTC 13031 (smooth variant)						
Drug	7H9 + 10% ADC + 0.05% Tween 80		ACFS + 0.05% tyloxapol		SCFM1 + 0.05% tyloxapol	
	MIC (μg/mL)	MBC (μg/mL)	MIC (μg/mL)	MBC (μg/mL)	MIC (μg/mL)	MBC (μg/mL)
LINEZOLID	6.25	>100	6.25	>100	25	>100
MINOCYCLINE	33.3	50	100	>100	41.6	>100
DOXYCYCLINE	100	100	100	>100	66.6	>100
AMIKACIN	6.25	12.5	0.781	8.3	0.781	10.4
CEFOXITIN	25	50	25	41.6	25	75
TIGECYCLINE	1.56	>100	1.56	>100	0.39	>100
IMIPENEM	3.125	>100	3.125	66.6	3.125	25
AZITHROMYCIN	25	16.6	>100	>100	100	>100
CLARITHROMYCIN	0.195	0.781	25	>100	6.25	25
MOXIFLOXACIN	0.391	>100	0.391	>100	0.391	>100

**Table 5:** Mean minimum inhibitory concentrations (MIC) and mean minimum bactericidal concentrations (MBC) of each drug in each media, against planktonic *M. abscessus* NCTC 13031 (rough variant)

M. *abscessus* NCTC 13031 (rough variant)						
Drug	7H9 + 10% ADC + 0.05% Tween 80		ACFS + 0.05% tyloxapol		SCFM1 + 0.05% tyloxapol	
	MIC (μg/mL)	MBC (μg/mL)	MIC (μg/mL)	MBC (μg/mL)	MIC (μg/mL)	MBC (μg/mL)
LINEZOLID	12.5	50	3.13	>100	50	>100
MINOCYCLINE	33.3	100	100	>100	50	>100
DOXYCYCLINE	16.6	33.3	33.3	>100	100	>100
AMIKACIN	12.5	12.5	0.781	66.6	0.781	66.6
CEFOXITIN	50	50	12.5	>100	12.5	>100
TIGECYCLINE	0.781	3.125	3.125	>100	0.39	25
IMIPENEM	3.13	12.5	1.56	29.1	0.78	50
AZITHROMYCIN	12.5	6.25	100	>100	>100	>100
CLARITHROMYCIN	1.04	0.781	50	50	25	50
MOXIFLOXACIN	0.781	4.16	6.25	>100	1.563	66.6

**Table 6:** Fold change in mean MIC for each antibiotic and species, in sputum media (ACFS/SCFM) compared to Middlebrook 7H9

	Fold change in mean MIC compared to 7H9									
	*M. abscessus* subsp. *abscessus*		*M. abscessus* subsp. *bolletii*		*M. abscessus* subsp. *massiliense*		*M. abscessus* NCTC 13031 (S)		*M. abscessus* NCTC 13031 (R)	
	SCFM	ACFS	SCFM	ACFS	SCFM	ACFS	SCFM	ACFS	SCFM	ACFS
**CLA**	170	4	1	>8	1	4	32	128	24	49
**TIG**	1	1	0.25	1	1	2	0.25	1	0.5	4
**MOX**	2	4	2	4	2	16	1	4	2	8
**AZI**	0.5	8	>8	>8	1	230	4	>4	>8	8
**IMI**	0.6	0.8	1	0.832	3	2.656	1	1	0.25	0.5
**LIN**	2	2	1	2	32	2	4	1	4	0.25
**AMK**	0.25	2	0.25	16	0.125	4	2	0.016	0.125	0.125
**MIN**	0.86	3.43	2	4	4	4	1	2	1.25	3
**CEF**	1	0.5	2	1	0.25	2	1	1	0.25	0.25
**DOX**	1	1	1	1	1.332	2	0.666	1	6	2

*AMK* amikacin, *AZI* azithromycin, *CEF* cefoxitin, *CLA* clarithromycin, *DOX* doxycycline, *IMI* imipenem, *LIN* linezolid, *MIN* minocycline, *MOX* moxifloxacin, *TIG* tigecycline.

**Table 7:** Fold change in mean MBC for each antibiotic and species, in sputum media (ACFS/SCFM) compared to Middlebrook 7H9

	Fold change in mean MBC compared to 7H9									
	*M. abscessus* subsp. *abscessus*		*M. abscessus* subsp. *bolletii*		*M. abscessus* subsp. *massiliense*		*M. abscessus* NCTC 13031 (S)		*M. abscessus* NCTC 13031 (R)	
	SCFM	ACFS	SCFM	ACFS	SCFM	ACFS	SCFM	ACFS	SCFM	ACFS
**CLA**	6.3	9.3	0.25	≥1	8	16	32	>128	64	64
**TIG**	1	1	1	1	5	>12	1	1	8	>32
**MOX**	8	32	>21.3	>21.3	8.03	>12.04	1	1	16	>24.038
**AZI**	>1.5	>1.5	1	1	<1	1	>6	>6	>16	>16
**IMI**	>0.5	>0.0625	0.44	0.38	0.798	9.62	0.666	>0.25	4	2.328
**LIN**	1	1	1	1	1	0.666	1	1	>2	>2
**AMK**	>9.6	4.8	10.1	34.1	1.39	>9.6	0.416	0.333	0.832	0.664
**MIN**	>3.43	>3.43	>1	>1	>1	>1	>2	>2	>1	>1
**CEF**	>4	>2.5	>3	3	0.5	0.75	1.5	0.832	>2	>2
**DOX**	1	1	1	1	0.666	1	>1	>1	>3	>3

*AMK* amikacin, *AZI* azithromycin, *CEF* cefoxitin, *CLA* clarithromycin, *DOX* doxycycline, *IMI* imipenem, *LIN* linezolid, *MIN* minocycline, *MOX* moxifloxacin, *TIG* tigecycline.

### Minimum inhibitory concentration

The impact of media type on MIC was largely strain and antibiotic dependent, with the differences largely depending on the combination in question. Overall, ACFS and SCFM1 mostly appear to induce decreases in MABSC antibiotic susceptibility, as evidenced by the increased MICs in these media types compared to 7H9 (Tables 1–5). A notable example of this is AZI against subsp. *bolletii*, whereby inhibitory activity was lost completely in both sputum medias despite being present at 12.5 μg/mL in 7H9 (Table 2 and Supplementary Fig. 1).

Although this trend is seen for most antibiotics, a few exceptions were observed whereby MIC was lower in either sputum media. One example includes CEF against subsp. *abscessus*, whereby the MIC was halved in ACFS compared to 7H9. Additionally, SCFM1 reduced the MIC of AMK by more than half compared to 7H9 across all subspecies.

When comparing the MIC in both SCFM1 and ACFS, 11/50 (22%) of conditions showed the same MIC; 29/50 (58%) showed a loss of susceptibility in ACFS; and the remaining 10/50 (20%) showed a reduced susceptibility in SCFM1. From this, we can determine that ACFS generally induces greater decreases in antibiotic sensitivity compared to SCFM1 when considering MIC.

### Minimum bactericidal concentration

Similarly, an increase in MBC was seen in the sputum medias compared to 7H9 for almost every antibiotic and isolate. Similar to the MIC findings, one exception that appeared to contradict this trend was CEF, with the mean MBC being lower in ACFS compared to 7H9 for NCTC 13031 smooth and subsp. *massiliense*. For the remaining three strains, however, CEF followed the same overall trend of reduced antibiotic susceptibility in the sputum media – especially prominent for NCTC 13031 rough whereby bactericidal activity was completely lost in SCFM1 and ACFS, despite being observed at 50 μg/mL in 7H9.

Comparison of MBC in either sputum media formulation revealed no difference in MBC for 48% (24/50) of the tested conditions. Additionally, 34% (17/50) of conditions showed a higher MBC in ACFS compared to SCFM1, and only 18% (9/50) demonstrated a higher MBC in SCFM1 versus ACFS. From this, we can interpret that ACFS generally induces greater reductions in antibiotic susceptibility compared to SCFM1 with regards to bactericidal activity.

## Discussion

Pathogens belonging to MABSC are incredibly difficult to treat, with their intrinsic resistance leading to treatment success as low as 45.6%^24^. This challenge is exacerbated by the standard drug screening assays used within antibiotic susceptibility testing, with the use of standard laboratory media providing a false predication of inhibitory antimicrobial concentrations in the lung. By poorly representing antimicrobial susceptibility, we raise the danger of exposing patients to subinhibitory antibiotic concentrations, unknowingly reducing compliance with antibiotic stewardship and perpetuating AMR when clinically implementing these in vitro results. Implementation of this strategy within research would additionally improve the accuracy of laboratory outcomes, thus helping to streamline hit identification. We therefore consider it vital that we improve the physiological relevance of our in vitro assays, to stand a better chance of overcoming this pathogen’s resistance in both the short and long term.

One proposed method of achieving this involves the application of sputum-mimetic media to conventional broth microdilution, with multiple sputum media formulations having been developed over time^13,14,16,18^. In this paper, we have applied two of these formulations to *M. abscessus* drug screening, using ten antibiotics representative of those that are clinically used to treat MABSC. Although strain and antibiotic dependent differences are seen, most antibiotics became less effective when cultured in either ACFS or SCFM1, with this difference being greater for ACFS. We believe that this better predicts the MIC and MBC exhibited in vivo, and thus it is unsurprising that these widely used antibiotics show poor success within the clinic.

Although yet to be evidenced, we hypothesise multiple reasons for the ACFS-induced reduction in antibiotic susceptibility encountered here. Interestingly, a change in cell physiology has been reported when *M. abscessus* is grown ACFS compared to 7H9, with an additional translucent sheath being demonstrated upon phase microscopy of ACFS-grown cells^17^. We believe that this sheath may be a contributing factor to the decreased antimicrobial susceptibility seen within ACFS, perhaps acting as a barrier to antimicrobial penetration further to its already highly impermeable cell wall. Furthermore, there is a potential for this resistance to be attributed to the composition of ACFS, with mucin and DNA previously being thought to influence drug pharmacokinetics^9^. Having a net negative charge^25^, mucins and DNA will bind to positively charged antimicrobials such as the aminoglycosides (AMK), perpetuating AMR^21,26^. Given that a higher AMK MBC is observed across the three subspecies when grown in ACFS (Fig. 2), we have strong rationale linking media formulation to AMK susceptibility. This evidence is compounded by the fact that mucin and DNA are absent from SCFM1 – a media type which induced less decreases in susceptibility compared to ACFS in most cases. To investigate the role of mucin and DNA within antimicrobial sensitivity, future work should involve modulation of these two media components, allowing any effects to be observed and directly correlated the presence of absence of mucin or DNA. Further work is therefore necessary to elucidate if there is any mechanistic link between this bacterial sheath or the media components, and the ACFS-induced reductions in sensitivity encountered here.

Although we believe this approach provides a significant step forward towards improving the predictability of in vitro drug screening, there are some factors that remain to be considered. For example, lung epithelial cells have been shown to influence antimicrobial susceptibility, which is a complexity that this work does not consider. When a dual-species biofilm of *M. abscessus* and *Pseudomonas aeruginosa* was grown on the human epithelial cell line A549, the activity of clarithromycin decreased; thought to be attributable the ability of this antibiotic to reside intracellularly. Intracellular uptake of clarithromycin into lung epithelial cells would decrease its bioavailability, in turn decreasing antimicrobial susceptibility. Thus, sputum media is not representative of the adverse lung environment on the whole^27^. This work is also restricted to individual antimicrobials rather than combinational regimens, which are typically employed clinically during the initial and continuation phases of MABSC treatment^22^. To better represent the clinical situation, future work should consider the impact of combining antibiotics in the same way that they are in vivo.

Furthermore, previous work has demonstrated that the MIC of multiple antibiotics in SCFM1 (including AMK, AZI, CEF and IMI) increases even further against biofilm-grown *M. abscessus* compared to planktonic alternatives—increasing more than 100-fold in some cases^28^. The same was observed for ACFS, with biofilm-associated cells being more antibiotic tolerant than planktonic cells taken from the same well^17^. Next steps for this work therefore include consideration of *M. abscessus* biofilms, with this aggregative phenotype being undeniably closer to that observed in vivo^29^. Further application of SCFM1 would enable us to maintain such physiologically relevant biofilm phenotypes, with the magnesium present within the medium being shown to facilitate biofilm formation^20^. Growth rate is also an important consideration, with MABSC being shown to grow more slowly in ACFS compared to 7H9 in previous studies—although the difference in doubling time is not statistically significant^17^. Since many antibiotics will target growth-essential functions (such as cell wall peptidoglycan synthesis, inhibited by the β-lactams cefoxitin and imipenem)^30^, differential growth rates may alter antibiotic activity. Further studies are necessary to remove the impact of growth rate, such as the use of colony counts that can be used to monitor bacterial growth simultaneously to antimicrobial activity. Colony counts would additionally enable more controlled enumeration of starting cell number, compared to the optical density adjustments used here.

Another development to this work should also include the consideration of polymicrobial infection, with one sole pathogen rarely being associated with CF. Rather, CF infections are typically caused by diverse polymicrobial communities, with one study isolating over 1800 different taxa from a cohort of CF patients^31^. It is known that members of these diverse communities can interact with one another; a process of which has been shown to influence antimicrobial susceptibility. For example, ceftazidime can inhibit a dual species biofilm of *M. abscessus* and *P. aeruginosa* better than a monomicrobial counterpart of *M. abscessus* alone^27,32^. It is therefore important to consider this layer of complexity during future research efforts, to better comprehend antimicrobial sensitivity in vivo. However, the diversity between each patient warrants a more personalised approach, which so far remains unattainable within medicine. We therefore argue that utilising sputum mimetic media offers a more feasible strategy to better understand MABSC antimicrobial susceptibility in vitro; a strategy that will ultimately improve clinical outcomes.

Overall, we have observed a decrease in MABSC antimicrobial susceptibility when the standard laboratory media 7H9 is replaced with the physiologically relevant sputum media ACFS and SCFM1. This difference was the most significant for ACFS, with the MIC and MBC being higher in ACFS compared to SCFM1 for 58% and 34% of the tested conditions, respectively. We therefore consider this data to justify a change in approach for in vitro culture, with the wider application of ACFS allowing better representation of in vivo drug susceptibility during in vitro antibiotic screening. Implementation of these physiologically relevant medias to routine testing is likely to drastically improve the accuracy of laboratory assays, thus improving treatment success and antibiotic stewardship when informing clinical decisions. Physiological relevance must therefore remain at the forefront of our future efforts to discover novel antimycobacterial compounds.

## Methods

### Bacterial isolates

The following strains were selected for study: *M. abscessus* NCTC 13031 (smooth and rough variant), 15944 subsp. *abscessus*, DC088A subsp. *bolletii* and DC088D subsp. *massiliense*. For the standard laboratory media condition, each species was routinely cultured in Middlebrook 7H9 broth containing 10% (v/v) Albumin-Dextrose-Catalase (ADC), 0.5% (v/v) glycerol and 0.05% (v/v) Tween80. Each species was cultured in SCFM1^33^ and ACFS^17^ for the physiologically relevant conditions, each with the addition of 0.05% (v/v) tyloxapol to facilitate the maintenance of planktonic culture. Both sputum medias were made as described previously^17,33^, with slight modifications to the sterilisation process for ACFS. Since protein functionality is lost when mucin is autoclaved^34^, porcine stomach mucin type II (Sigma) was sterilised using 70% ethanol. Mucin powder was placed into 70% ethanol at a concentration of 100 mg/mL^35^ and heated to 60 ^o^C for 2 h. This was left overnight at 25 ^o^C and centrifuged at 500 × *g* for 10 min the following day. The ethanol supernatant was poured off, and the remaining mucin pellet was added to the remainder of the filter sterilised ACFS media. All cultures were incubated at 37 °C with orbital shaking at 180 rpm for 72 h.

### Antimicrobials

Tetracycline (TET), minocycline hydrochloride (MIN) and doxycycline hyclate (DOX) were sourced from Sigma Aldrich (Dorset, UK). Imipenem (IMI), cefoxitin (CEF), azithromycin (AZI), tigecycline (TIG), linezolid (LIN) and clarithromycin (CLA) were sourced from Carbosynth (Compton, UK). Amikacin (AMK) was sourced from Melford Laboratories (Suffolk), and moxifloxacin hydrochloride (MOX) from Santa Cruz Biotechnology (Dallas, Texas).

Stock solutions of all compounds were prepared in DMSO, apart from DOX, AMK and IMI which were prepared in sterile dH_2_O. All stocks were stored at –20 ^o^C excluding IMI, which was made fresh for each use to maintain stability.

### Broth microdilution assays

Antibiotics were serially diluted twofold in 96-well microtiter plates, at a final concentration of 100 μg/mL to 0.390625 μg/mL. At the end of each dilution series, a 0 μg/mL control was incorporated by using DMSO or dH_2_O only. In each test well, 99 μL of bacterial culture adjusted to OD_600nm_ = 0.1 was added (*n* = 3). Appropriate positive and negative controls were incorporated via the inclusion of 100 μL of bacteria and media only (*n* = 8), respectively. When the minimum inhibitory concentration (MIC) fell below 0.390625 μg/mL, these assays were repeated with a lower concentration range (twofold serial dilutions of 0.39 μg/mL to 0.001526 μg/mL).

Plates were incubated statically at 37 ^o^C in aerobic conditions for 96 hours^36,37^. Absorbance (570 nm) was read at 0 h and 96 h on a spectrophotometric plate reader (BioTek 800 TS) to monitor the degree of bacterial growth. Subsequently, 30 μL 0.1% (w/v) resazurin substrate solution was added to each well and the plates were incubated in the same conditions for a further 24 h. The lowest concentration at which a blue colour was observed indicated the minimum inhibitory concentration (MIC), with the presence of viable microorganisms below the MIC causing resazurin reduction and a corresponding blue-to-pink colour change.

### Minimal bactericidal concentration

After 96 h of incubation, the contents of each well were stamped onto square Middlebrook 7H11 agar plates supplemented with 0.5% (v/v) glycerol, to facilitate the measurement of minimum bactericidal concentration (MBC). Spots were dried in a biological safety cabinet prior to incubation at 37 ^o^C for 96 h. MBC was ascertained from the lowest drug concentration where no bacterial growth occurred.

### Data analysis

MIC and MBC data was gathered by visual observation, and the mean value plotted as bar graphs on GraphPad Prism 8 (*n* = 3) (Figs. 1, 2). To consider the visualisation capacity of different people, validation of MIC was carried out by a second individual. Calculation and plotting of the mean values allowed for any outliers to be taken into consideration.

## Supplementary information

Supplementary information

## Data Availability

The datasets used and/or analysed during the current study are available from the corresponding author on reasonable request.
