# Supplementary material for: Cystic fibrosis sputum media induces an overall loss of antibiotic susceptibility in Mycobacterium abscessus
Source: NPJ Antimicrob Resist. 2024 Nov 5;2:34. doi: 10.1038/s44259-024-00054-3 (PMC11721417; doi:10.1038/s44259-024-00054-3)
Supplement: Supplementary file 1 — Supplementary information [file 44259_2024_54_MOESM1_ESM.pdf]

Supplementary Information:

Supplementary Figure 1: Heat maps demonstrating the minimum inhibitory concentration (MIC) and minimum bactericidal concentration (MBC) of each antibiotic in each media, against the five strains of *M. abscessus*. Light to dark colouration indicates the most to least susceptible, respectively. AMK, amikacin; AZI, azithromycin; CEF, cefoxitin; CLA, clarithromycin; DOX, doxycycline; IMI, imipenem; LIN, linezolid; MIN, minocycline; MOX, moxifloxacin; TIG, tigecycline.

MEAN MIC (µg/mL) COLOUR SCALES

M. abscessus subsp. *abscessus*

	LIN	MIN	DOX	AMK	CEF	TIG	IMI	AZI	CLA	MOX
**7H9**	3.13	14.58	50	6.25	25	0.78	2.6	1.563	0.098	0.7813
**SCFM1**	6.25	12.5	50	1.563	25	0.78	1.56	0.781	16.6	1.5625
**ACFS**	6.25	50	50	12.5	12.5	0.78	2.08	12.5	1.563	3.125

M. abscessus subsp. *bolletii*

	LIN	MIN	DOX	AMK	CEF	TIG	IMI	AZI	CLA	MOX
**7H9**	25	25	50	0.391	25	1.56	6.25	12.5	12.5	1.5625
**SCFM1**	25	50	50	0.098	50	0.39	6.25	>100	12.5	3.125
**ACFS**	50	100	50	6.25	25	1.56	5.2	>100	>100	6.25

M. abscessus subsp. *massiliense*

	LIN	MIN	DOX	AMK	CEF	TIG	IMI	AZI	CLA	MOX
**7H9**	3.13	25	50	25	25	0.78	3.13	0.195	0.098	1.5625
**SCFM1**	100	100	66.6	3.125	6.25	0.78	9.38	0.195	0.098	3.125
**ACFS**	6.25	100	100	50	50	1.56	8.3	50	0.391	25

M. abscessus NCTC 13031 smooth

	LIN	MIN	DOX	AMK	CEF	TIG	IMI	AZI	CLA	MOX
**7H9**	6.25	50	100	6.25	25	1.56	3.13	25	0.195	0.391
**SCFM1**	25	50	66.6	0.098	25	0.39	3.13	100	6.25	0.391
**ACFS**	6.25	100	100	12.5	25	1.56	3.13	>100	25	0.391

M. abscessus NCTC 13031 rough

	LIN	MIN	DOX	AMK	CEF	TIG	IMI	AZI	CLA	MOX
**7H9**	12.5	33.3	16.6	6.25	50	0.78	3.13	12.5	1.04	0.7813
**SCFM1**	50	41.6	100	0.781	12.5	0.39	0.78	>100	25	1.5625
**ACFS**	3.13	100	33.3	0.781	12.5	3.13	1.56	100	50	6.25

MEAN MBC (µg/mL) COLOUR SCALES

M. abscessus subsp. *abscessus*

	LIN	MIN	DOX	AMK	CEF	TIG	IMI	AZI	CLA	MOX
**7H9**	>100	29.16	>100	10.42	16.6	>100	>100	66.6	0.098	3.125
**SCFM1**	>100	>100	>100	>100	66.6	>100	50	>100	0.618	25
**ACFS**	>100	>100	>100	50	41.6	>100	6.25	>100	0.911	100

M. abscessus subsp. *bolletii*

	LIN	MIN	DOX	AMK	CEF	TIG	IMI	AZI	CLA	MOX
**7H9**	>100	100	>100	1.953	33.3	>100	66.6	>100	>100	4.6875
**SCFM1**	>100	>100	>100	19.79	>100	>100	29.2	>100	25	>100
**ACFS**	>100	>100	>100	66.6	100	>100	25	>100	>100	>100

M. abscessus subsp. *massiliense*

	LIN	MIN	DOX	AMK	CEF	TIG	IMI	AZI	CLA	MOX
**7H9**	>100	100	>100	10.42	66.6	8.3	10.4	>100	0.098	8.3
**SCFM1**	>100	>100	66.6	14.58	33.3	41.6	8.3	100	0.781	66.6
**ACFS**	66.6	>100	>100	>100	50	>100	>100	>100	1.563	>100

M. abscessus NCTC 13031 smooth

	LIN	MIN	DOX	AMK	CEF	TIG	IMI	AZI	CLA	MOX
**7H9**	>100	50	100	37.5	50	>100	>100	16.6	0.781	>100
**SCFM1**	>100	>100	>100	1.563	75	>100	25	>100	25	>100
**ACFS**	>100	>100	>100	12.5	41.6	>100	66.6	>100	>100	>100

M. abscessus NCTC 13031 rough

	LIN	MIN	DOX	AMK	CEF	TIG	IMI	AZI	CLA	MOX
**7H9**	50	100	33.3	12.5	50	3.13	12.5	6.25	0.781	4.16
**SCFM1**	>100	>100	>100	10.4	>100	25	50	>100	50	66.6
**ACFS**	>100	>100	>100	8.3	>100	>100	29.1	>100	50	>100

ANTIBIOTIC CONCENTRATION (µg/mL)

- 0.098
- 0.195
- 0.391
- 0.781
- 1.563
- 3.125
- 6.25
- 12.5
- 25
- 50
- 100
- >100
